# Importance of an International Registry for and Collaborative Research on Esophageal Atresia

**DOI:** 10.3389/fped.2017.00081

**Published:** 2017-04-20

**Authors:** Frédéric Gottrand, Delphine Ley, Laurent Michaud, Rony Sfeir

**Affiliations:** ^1^Reference Center for Congenital and Malformative Esophageal Disorders, CHU Lille, Univ. Lille2, Lille, France

**Keywords:** esophageal atresia, congenital defect, international registry, population-based cohort, collaborative research

## Abstract

Esophageal atresia (EA) is a rare congenital defect. Data on EA prevalence, management, and long-term outcome are lacking because the available data come from small retrospective series from tertiary referral centers. An international multicenter registry would provide strong epidemiological data from large population-based cohorts on EA prevalence and incidence, treatment, long-term morbidity, and prognosis and would thus provide accurate data for evaluation of the current guidelines for EA management. The future challenge of the new international network on EA, which was created in 2013, is to promote the creation of a collaborative database and further studies.

## Current Level of Evidence about Esophageal Atresia (EA)

Esophageal atresia is a rare congenital anomaly whose origin remains unknown. Most available studies on EA are small retrospective series from tertiary centers, and the quality of the data remains limited because of the low statistical power and selected population. Moreover, the results cannot be extrapolated to the general population of EA patients. At the recent international conference on EA organized by the International Network on Esophageal Atresia (INoEA) in Sydney, Australia,^1^ only 14 of 76 selected abstracts were multicenter studies. However, the number of patients included in these 14 collaborative studies (*n* = 2,238) exceeded the total number of patients included in the other 62 single-center studies (*n* = 1,901).

When preparing a consensus statement on the available evidence about EA, a systematic literature search was performed using the classification system of the Oxford Centre for Evidence-Based Medicine and Grade evidence profile. Although the importance was classified as “critical” for most of the 167 references selected for this consensus, only 29 were classified as providing high-quality evidence, 18 were classified as providing moderate evidence, and 130 were qualified as providing a low or very low quality of evidence (1). As a consequence, all statements and recommendations on EA available today are opinion based (i.e., all of the 40 statements in the consensus mentioned above) (1). Even now, collaborative studies on EA remain rare. At the end of January 2017, only 10 studies on EA were registered in the Clinical Trials registry.

## Current Registries and Databases for EA

The prevalence of EA has been established from global birth surveillance programs (network of malformation registers) throughout the world (2). Data from these programs (EUROCAT in Europe and the National Birth Defects Prevention Network in the USA) suggest that the prevalence of EA is similar between countries and stable over time (3). However, because these registers focus on prenatal and neonatal diagnosis of several malformations, they include limited details about neonatal treatment and no information about the early or late outcome of EA patients (4, 5). There are recent initiatives to set up specific population-based EA registers at a country level (e.g., Australian and French registers) (6, 7). The advantages of these registers are that they are population based and can provide precise and detailed information about EA and early outcomes (8). As for other rare diseases, one of the main gaps in understanding and treating EA is the lack of in-depth knowledge about the natural history of the malformation, which is one prerequisite for undertaking clinical trials.

## International Collaborative Registries

There are arguments for and against multicenter studies (Table 1). One argument is that there is a need to set up multicenter studies to include a large number of EA patients to answer the many questions about the impact of prenatal diagnosis on the outcome and live birth prevalence, optimal surgical treatment for long-gap EA, treatment of gastroesophageal reflux disease, and risk for cancer over the long term. The positive aspects of collaborative efforts at the international level include providing strong epidemiologic data, monitoring the birth prevalence of EA, detecting new or continuing trends, identifying new potentially teratogenic exposures, and evaluating the effects of different prenatal policies. Such registers also provide a unique opportunity to set up prospective population-based cohort studies, nested case–control studies, and case–cohort design studies. Furthermore, multicenter studies may provide unique information for health authorities about the prevalence, long-term morbidity, and disabilities of EA, which will help in determining health policy priorities. These rare disease registries are also an opportunity to pool data from many centers to achieve a sufficient sample size for epidemiological and clinical research. They will also be useful for assessing the feasibility of clinical trials, planning appropriate clinical trials, and supporting patient enrollment.

**Table 1:** **Arguments for and against multicenter studies of esophageal atresia (EA)**.

Opportunities	Limitations
High power of studies (stratification/subgroup analysis) Population-based studies vs. highly selected population No other option for some rare forms of EA (i.e., long gap) Sharing knowledge, harmonization of care Collaborations between centers/countries Evaluation of the recommendations Better acknowledgment (health authorities, scientific societies, journals) Special funding for rare diseases	Long process Differences in ethical/regulatory rules between countries Need to harmonize care Need to harmonize data to be collected (questionnaire) Difficulty in achieving and maintaining quality control of data Need for support (research personnel, database manager) Authorship Higher cost/lack of funding

One example of such a network is the recently reported successful establishment of a multicenter network for hepatoblastoma. The development of biological markers for this very rare tumor and identification of reliable prognostic risk factors for tailoring treatment remain challenging. The consortium comprises the four multicenter trial groups that have performed prospective controlled studies on hepatoblastoma over the past two decades. A centralized online platform has been created in which data from eight completed hepatoblastoma trials have been merged to identify prognostic factors and to confirm existing established factors (9). There is also a successful initiative for multicenter studies of some forms of neuronal ceroid lipofuscinoses (10). These examples show the value of compiling, in a single database, the natural history data available in different countries, clinics, research institutions, and families.

The PedNet registry is a multicenter observational research database for hemophilia. All patients with hemophilia born after January 1, 2000, and treated in 1 of the 29 participating hemophilia treatment centers are included (11). All centers prospectively collect data, including treatments and outcomes, for all included patients. By using the data from this database, two major studies compared the effects of the type of factor concentrate and prophylaxis treatment vs. on-demand treatment on inhibitor development in severe hemophilia (11). The observational cohort design does not interfere with the day-to-day clinical management of patients and improves the clinical management over time. The monitoring of centers in this cohort by experienced study coordinators has been shown to be critically important for improving the quality of the data. It is important to note that all patients from the participating centers must be included to prevent selection bias (11).

There is a recent initiative of the European Union to set up a European platform for rare disease registries. The main objectives of this platform are to provide a central access point for information on registries of patients with rare diseases for all stakeholders and to support existing registries in view of their interoperability. However, international registries and databases have constraints and disadvantages when compared with national registries or databases, such as interoperability problems, cost, and regulations. Another possible disadvantage of collaborative registries is the lack of exhaustiveness. The Children’s Cancer Research Network was created to explore the epidemiological landscape of rare childhood cancers in the USA and Canada. In addition to poor registration rates, tissue samples of these cancers were scarce, and tissues for banking were submitted for only 11% of all cases of rare tumors in this registry (12).

The necessary studies would be easier to conduct within the context of a unique homogeneous database or interoperable national databases. Building this kind of structure would encourage every participating state to encourage local and national authorities to create a national rare disease database or specific EA atresia register. For all such databases or registries, it is essential that the data should be sharable. A future international database setting would have many advantages (Table 1), the most important being the homogeneity of data. Having these data in a centralized register could provide greater visibility for future research projects, even for the smallest group of patients (stratification), which would allow comparisons of results and techniques within and between homogenous groups of patients (e.g., type A EA, esophageal replacement techniques) and the wide distribution of findings.

## Necessary Factors for Creating a Successful International Collaborative Registry for EA

There is a movement toward increasing research collaboration, greater data sharing, and increasing engagement and active involvement by patients, advocates, and foundations for rare diseases (13). The growth in networks and social networking tools presents opportunities to help reach other patients, to find researchers, and to build collaborations. Engagement of patients’ and parents’ associations with other stakeholders in clinical research may help to ensure that research efforts related to rare diseases address the relevant clinical questions and provide patient-centered health outcomes. Rare disease organizations may provide an effective means of facilitating patient engagement in research (14). The success of such approaches and common challenges inherent in directly engaging patients, patient advocacy groups, and investigators in the creation, growth, and productivity of multicenter research groups involved in clinical research on rare diseases has been reported (15). From this perspective, the INoEA collaboration with the international federation of patient support groups (EAT)^2^ provides opportunities for collaborative studies on EA through joint lobbying, communication, and fundraising. The recent appointment by the European Commission of a European reference network for rare digestive diseases and malformation^3^ may also provide an opportunity to structure international collaborations for research on EA.

Another example is Castleman’s disease, a very rare disease whose cause and pathogenesis of the idiopathic form are unknown. Researchers studying the idiopathic form have never received National Institutes of Health funding, and a single disease research organization has been the only source of research funding. The small sample sizes at individual institutions have prevented research from being adequately powered to test for significance. In 2012, the Castleman’s Disease Collaborative Network (CDCN) was created by assembling a group of physicians, researchers, and patients to create and accelerate research through a targeted, collaborative, and patient-centric approach. Their aims are first to build a global community, to leverage the community to prioritize studies and share research samples, and to fund community-prioritized research by seeking proposals and funding strategically directed research grants to experts (16). In the past year, the CDCN has supported more than 6,000 patients and support groups through an online patient forum, patient summit, and website. Engagement of patients in the research process has aligned stakeholders’ incentives to conduct research that will have the greatest impact on patients’ lives (16).

The Consortium for Clinical Investigations of Neurological Channelopathies and the Clinical Research Consortium for Spinocerebellar Ataxias engage patients with rare neurological channelopathies with investigators and with advocacy groups in multicenter networks (17). These two networks have created patient registries, stratified on the basis of genetic characteristics, and included longitudinal clinical data. By using these patient registries, disease-relevant outcome measures have been identified. Moreover, phase I and II trials have been conducted by the networks. Patient advocacy groups provide essential support for networks by providing financial and logistical support for research activities, such as organizing patient registries and investigator meetings (17).

The informed consent process is a challenge to sharing data among research consortia and adds a layer of complexity that requires coordination between research centers worldwide. Rare disease consortia face specific challenges because the available data and samples may be very limited. Therefore, it is especially relevant to ensure the best use of available resources but at the same time to protect patients’ right to integrity. Achieving this aim requires the ethical duty to plan in advance the best possible consent procedure to address the potential ethical and legal hurdles that could hamper research in the future. It is especially important to identify the key core elements to be addressed in informed consent documents for international collaborative research in two different situations: (i) new research collections (biobanks and registries) for which information documents can be created according to current guidelines and (ii) established collections obtained without informed consent or with previous consent that does not cover all key core elements (18).

Another challenge is the standardization of definitions and data collection (19). Uniform approaches are necessary for robust collaborative research, particularly involving rare diseases. Collaborative research involving multiple centers and groups requires critical procedures to be coordinated to facilitate accurate comparisons of data. The use of standard operating procedures for the collection and handling of samples and data is a critical first step in ensuring high-quality translational research (19). Disseminating such information among researchers requires a flexible and secure data-sharing infrastructure (19).

The specific goals of an international registry of EA should include epidemiological surveillance of the malformation, first-year outcome, prospective population-based cohort studies, and nested case–cohort studies. The resources needed would include coordinators to define the minimal data set and interoperability, to standardize and monitor the quality of the data, and to develop a centralized database platform. Recent international or government initiatives on rare diseases (e.g., the recently launched European networks of reference for rare diseases) may represent a unique opportunity to achieve these goals within a realistic time frame.

## Conclusion

To face these challenges, the international network on EA, INoEA, was created in 2013. The INoEA is an informal multidisciplinary group of clinicians, researchers, allied health professionals, and family support group representatives who have joined efforts to improve research and care for EA patients. The goals of INoEA are to favor collaboration and to share information between centers throughout the world. Initiating a collaborative database and further studies are challenges for the future.

## Author Contributions

All authors contributed to the drafting and critical revision of the work. All authors have approved the final version of this manuscript.

## Conflict of Interest Statement

The authors declare that the research was conducted in the absence of any commercial or financial relationships that could be construed as a potential conflict of interest. The handling editor declared a past co-authorship with one of the authors, FG, and states that the process nevertheless met the standards of a fair and objective review.

## Footnotes

^1^http://www.oa2016.com.au.

^2^www.we-are-eat.org/.

^3^http://ec.europa.eu/health/rare_diseases/european_reference_networks/erf_en.
